# Navigating Calciphylaxis in End-Stage Renal Disease: A Case Report Highlighting the Complexities of Pain Control and the Need for Greater Clinical Vigilance

**DOI:** 10.7759/cureus.96962

**Published:** 2025-11-16

**Authors:** Ryan Ching Nam Lau, Bethan Laura Whitwell, Christma Chinawanitkit, Farhaan Ahmed

**Affiliations:** 1 Renal Medicine, Queen Elizabeth Hospital Birmingham, Birmingham, GBR

**Keywords:** calciphylaxsis, end-stage renal disease, high bmi, pain management, sodim thiosulfate, systemic steroids, warfarin

## Abstract

We present this case to increase awareness of calciphylaxis to promote early diagnosis and, therefore, improved clinical outcomes. Calciphylaxis is a rare, life-threatening complication of end-stage renal disease (ESRD) characterised by vascular calcification, thrombosis, and skin necrosis. We describe a 48-year-old female on long-term haemodialysis with obesity and prior warfarin use who developed painful abdominal plaques progressing to necrotic ulcers over six weeks. A skin biopsy performed at seven weeks confirmed focal calciphylaxis. Management involved intensified haemodialysis, intravenous sodium thiosulfate (12.5 g five times a week), and switching from warfarin to apixaban (2.5 mg twice daily). Despite specialist input and multimodal analgesia with opioids and adjuvant agents, pain control remained extremely challenging and significantly impacted quality of life. The case highlights the need for early recognition, prompt multidisciplinary management, and greater awareness among healthcare professionals of the severe pain burden and high morbidity associated with calciphylaxis to facilitate timely diagnosis and improve patient outcomes.

## Introduction

Calciphylaxis is a rare but potentially life-threatening condition most commonly associated with end-stage renal disease (ESRD). It is characterised by calcium deposition within the small and medium-sized blood vessels of the skin and subcutaneous tissue. This process leads to painful skin lesions and tissue necrosis, typically affecting the adipose-rich areas of the trunk and extremities. The underlying pathogenesis is multifactorial. It involves dysregulation of calcium and phosphate metabolism and abnormal parathyroid hormone (PTH) levels. These changes, particularly hyperphosphatemia, promote the transformation of vascular smooth muscle cells (VSMCs) into osteoblast-like phenotypes, leading to vascular calcification [1]. The concomitant vascular calcification and hypercoagulability contribute further to ischemic necrosis [1]. Therefore, the development of skin necrosis in a patient receiving warfarin should raise clinical suspicion for calciphylaxis.

Although calciphylaxis can develop in individuals with normal renal function, the majority of cases occur in patients with advanced kidney disease [2]. Studies have reported a rising incidence over the last decade, with estimates ranging from 0.04% to 4% among dialysis-dependent patients [3]. Patients with calciphylaxis have a markedly worse prognosis than dialysis recipients without the condition; a UK-based study reports an annual mortality rate of approximately six-fold increase compared with matched dialysis controls [4]. While the six-month mortality rate is approximately 33% in patients who presented with plaques only, it increases significantly to over 80% once ulceration develops [5], with the most common cause of death being sepsis from infected ulcers [2]. Several risk factors have been associated with calciphylaxis, including female sex, obesity, diabetes mellitus, hypoalbuminaemia, autoimmune conditions, malignancy, warfarin, corticosteroid use, and calcium-based phosphate binders, among others [2]. In this case, the patient is a female with a high body mass index (BMI), on warfarin, with a history of ESRD on haemodialysis, thus encompassing multiple strong risk factors for calciphylaxis.

High BMI presents a fourfold increased risk for calciphylaxis, likely a result of chronic tension of subcutaneous septa and arterioles produced by excess adipose tissue, combined with oedema [6]. The exact reason for the increased risk in females is unknown. Vitamin K antagonists, such as Warfarin, are important risk factors, as they inhibit the enzyme responsible for activating matrix Gla protein (MGP), a vitamin K-dependent protein involved in the inhibition of calcification [7,8]. It is through this mechanism that these drugs are thought to contribute to the development of calciphylaxis and should therefore be avoided in high-risk patients (although this may be challenging due to limitations in safe anticoagulation in ESRD patients). Research showed that between 40% and 50% of patients with calciphylaxis are reported to be on vitamin K antagonists, such as warfarin, at the time of presentation [7]. In this case, the patient was already established on warfarin; therefore, in light of the working diagnosis, it was switched to apixaban. Autoimmune diseases such as Sjogren’s may also pose a strong risk factor for the development of calciphylaxis in non-dialysis patients, although this is not applicable in this case.

This case report aims to contribute to the current understanding of calciphylaxis and emphasise the complexities of its multidisciplinary management, particularly in relation to pain management, to improve patient experience and clinical outcomes.

## Case presentation

**Figure 1:**
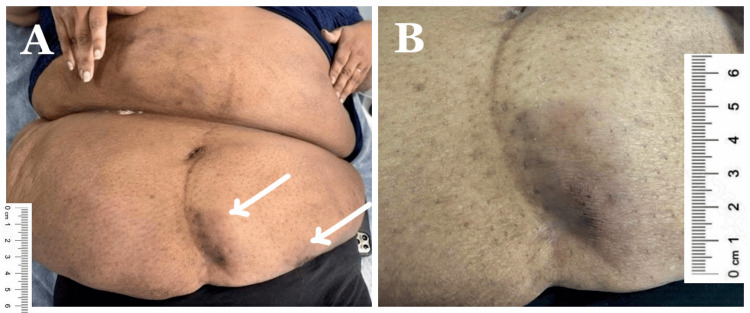
Lower abdominal subcutaneous plaques at three weeks of presentation with overlying discolouration and early nodules (arrows in A). (B) A close-up view of a firm, indurated subcutaneous plaque measuring 5 cm x 2.5 cm. Photos were taken with patient consent.

A 48-year-old female was admitted to a tertiary renal unit from the community dialysis centre due to a two-month history of an increasing number of abdominal plaques with associated severe pain. She has a background of chronic kidney disease (CKD) secondary to obstructive nephropathy caused by bilateral retroperitoneal fibrosis. She has previously undergone a nine-month trial of corticosteroid therapy, which resulted in significant weight gain, and has been maintained on haemodialysis for the past eight years. Her past medical history includes chronic back pain, a high body mass index (BMI) of 55 and hypertension. The patient noted firm plaques over the lower abdomen that progressively increased in number over the following weeks. Three weeks after the onset of presentation, one of the plaques measured 5 cm x 2.5 cm with discolouration and associated pain (Figure 1). During the initial review, the lesions were presumed to be lipomas, and no investigations were performed. Over the next two weeks, the plaques rapidly progressed, becoming indurated and erythematous, with some developing superficial ulceration and exudative discharge. The patient reported severe, debilitating pain that significantly limited daily activities. Due to rapid clinical deterioration, she was admitted seven weeks from symptom onset for further diagnostic evaluation, optimisation of pain management and multidisciplinary assessment.

**Figure 2:**
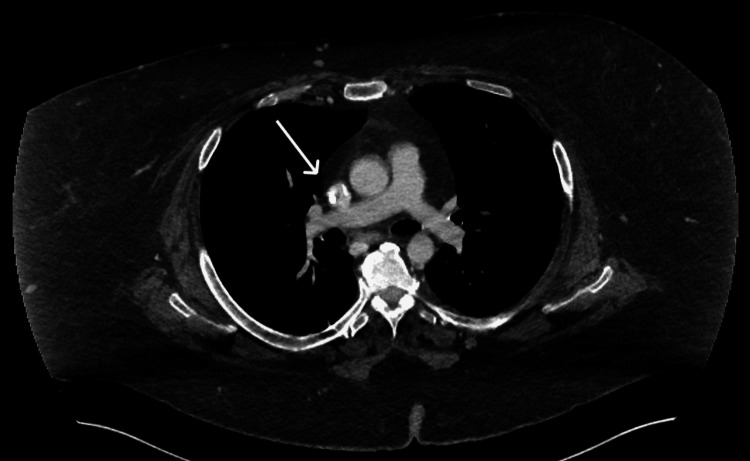
Axial contrast-enhanced CT image of the thorax demonstrating dense coronary artery calcifications marked by an arrow

**Figure 3:**
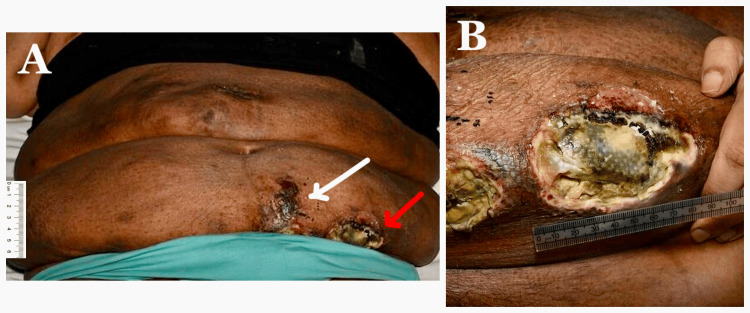
Extensive lower abdominal necrotic ulcerations at sites of previous plaques eight weeks into admission, with surrounding erythema and induration (arrows in A). (B) One of the chronic ulcers with a necrotic base and irregular, undermined margins, measuring approximately 5 cm × 8 cm.

Upon hospital admission, the patient underwent a series of investigations to establish a diagnosis and guide management. Her weight was 128 kg, reflecting a 40 kg loss over the previous 18 months with the use of tirzepatide. Given the patient’s significant weight loss, in conjunction with the new clinical suspicion of calciphylaxis in the context of ESRD, a computed tomography (CT) scan of the thorax, abdomen and pelvis was performed. The imaging revealed no evidence of primary or metastatic thoracoabdominal malignancy, but demonstrated moderately severe coronary artery calcification (Figure 2). Blood tests revealed elevated creatinine, raised C-reactive protein (CRP) and increased alkaline phosphatase (ALP), with normal phosphate and corrected calcium levels (Table 1). An incisional skin biopsy, the gold standard for confirming the diagnosis in patients presenting with characteristic skin lesions [1,4], was performed at seven weeks of symptom onset. Histopathology demonstrated florid fat necrosis with patchy chronic inflammation and focal medial arteriolar calcification, consistent with focal calciphylaxis. Nevertheless, based on the overall clinical presentation, a diagnosis of calciphylaxis was made. Eight weeks into admission, extensive necrotic ulcerations with surrounding erythema, induration and purulent discharge were present over the lower abdomen at the sites of prior plaques (Figure 3), where one of the chronic ulcers measured 5 cm x 8 cm, indicating progressive disease with poor wound healing.

**Table 1:** Blood test results on admission.

Test	Result	Normal reference range	Interpretation
Urea	4.0 mmol/L	2.5-7.8 mmol/L	Within normal range
Creatinine	367 µmol/L	60-110 µmol/L	Significantly elevated
Albumin	24 g/L	35-50 g/L	Low
Corrected calcium	2.34 mmol/L	2.2-2.6 mmol/L	Within normal range
Phosphate	1.12 mmol/L	0.8-1.5 mmol/L	Within normal range
Alkaline phosphatase (ALP)	466 U/L	30-130 U/L	Significantly elevated
C-reactive protein (CRP)	230 mg/L	<5 mg/L	Significantly elevated
Haemoglobin (Hb)	101 g/L	120-160 g/L (female)	Low
White cell count (WCC)	8.49 x 10^9^/L	4.0-11.0 x 10^9^/L	Within normal range

Current management strategies for calciphylaxis usually involve decalcification, wound care and pain control [7]. In this case, the patient’s haemodialysis regimen was intensified from three to five sessions per week, and intravenous sodium thiosulphate (STS) at a dose of 12.5 g administered post-dialysis was initiated, five times weekly. STS therapy was commenced six weeks after the onset of presentation, as part of standard therapy for calciphylaxis. The patient experienced nausea as a side effect, which subsequently improved with the introduction of antiemetic medication; no other side effects were reported. STS binds to calcium to form the more soluble calcium thiosulphate, thereby reducing calcium deposition [9]. The patient had been receiving warfarin for a previous pulmonary embolism (2021) and recurrent clotted vascular graft. However, given the recognised association between warfarin therapy and the development of calciphylaxis, warfarin was discontinued and subsequently switched to apixaban 2.5 mg twice daily to reduce the risk of further vascular calcification while maintaining protection against thromboembolic events. Pain management proved challenging, as severe, disproportionate pain is characteristic of calciphylaxis. The patient was initially reviewed by the acute pain team due to the excruciating pain associated with her necrotic skin lesions, followed by the chronic pain service. Buprenorphine 5 mcg/hour patch was commenced and up-titrated to 10 mcg/hour, alongside maximal-dose tramadol, but due to persistent severe pain, multiple opioid regimens were trialled, including fentanyl (patch and lozenges) and both immediate- and sustained-release oxycodone. 

**Figure 4:**
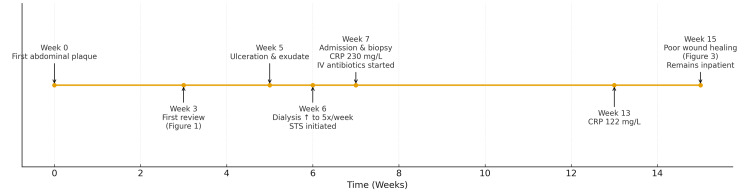
Timeline of main events relevant to this case. Image credit: Ryan Ching Nam Lau CRP, C-reactive protein; IV, intravenous; STS, sodium thiosulphate

Pain management remained challenging for this patient. Following the escalation of high-potency opioids and adjunct neuropathic analgesics, the patient developed visual and auditory hallucinations as symptoms of opioid toxicity. Opioid reversal agent (Naloxone 400 and 800 mcg boluses) was administered; however, she remained drowsy and confused and subsequently became haemodynamically unstable. Haemodynamic stability was achieved with fluid resuscitation, and a trial of intravenous corticosteroids was initiated. Her analgesic regimen was then reviewed and rationalised to prevent further opioid-related toxicity. The admission pain score was 9/10, which improved to 4/10 six weeks into admission. Furthermore, the patent’s wound cultures isolated multiple organisms, including *Streptococcus sanguinis*, *Actinomyces oris* and *Cupriavidus gilardii*, each with varying antimicrobial sensitivities. Blood cultures were obtained regularly, but did not grow any organisms. Following microbiology input, the patient was treated with a combination of intravenous teicoplanin (maintenance dose of 1,440 mg every 72 hours), ertapenem (1 g three times a week) and gentamicin (240-360 mg every 72 hours). Despite more than six weeks of intravenous antibiotic therapy, her CRP level decreased from 230 to 122 mg/L; however, wound healing remained minimal. She remains an inpatient under multidisciplinary management to support ongoing wound care, pain control and discharge planning. The timeline of events is summarised in Figure 4.

## Discussion

Calciphylaxis is a rare, rapidly progressing disease, with an estimated one-year survival rate reported to be between 45% and 55% [10]. It is estimated to have an incidence of 0.04% to 4% among dialysis-dependent patients [3], limiting opportunities for large-scale clinical studies and hindering research and funding. Its pathophysiology encompasses dysregulation of calcium and phosphate metabolism, vascular calcification, atherosclerotic plaques and hypercoagulability [1]. The multifactorial complexity of calciphylaxis required input from a number of specialities to ensure optimal outcomes. In this case, there was involvement of nephrology, dermatology, microbiology, tissue viability, chronic pain and acute pain teams. Nephrology remained the parent team throughout the admission and coordinated patient management. This involved liaising with the dialysis unit, prescribing appropriate treatment, such as sodium thiosulphate, and coordinating input from other specialities. Dermatologists were responsible for the biopsy of the lesions, which a formal diagnosis necessitated, and contributed to wound care advice alongside tissue viability specialists. Chronic and acute pain teams were critical in maintaining patient comfort. This case highlights the importance of a collaborative, multidisciplinary approach in the management of certain diseases. The involvement of multiple specialities was imperative in providing suitable care, as the condition could not be managed by a single team alone. The input from the pain team was essential in patient comfort, whilst the renal and dermatology teams were involved in the initial diagnosis and subsequent treatment. This reinforces the role of multidisciplinary team discussions in achieving optimal patient outcomes, emphasising the importance of coordinated input from a variety of disciplines. 

Pain management remains a key component in the management of calciphylaxis. Achieving good control of pain can be challenging, owing to both the intense pain of the lesions, but also the additional complexity of renal impairment, limiting analgesic choice in the majority of calciphylaxis patients. Fentanyl and methadone are usually preferred, as they lack active metabolites that accumulate in renal failure [7]. The efficacy of pharmacological and non-pharmacological interventions varies greatly between patients [11]. In this case, the patient was initiated on as-needed tramadol, alongside a buprenorphine patch (after an initial trial on fentanyl patches had been unsuccessful). Despite maximum dosing, pain was still becoming increasingly challenging to manage, especially during dressing changes and mobilisation; therefore, advice was sought from pain specialists. Under the guidance of the chronic pain team, tramadol was later replaced by oxycodone due to ongoing unbearable pain. Fentanyl lozenges were also successfully trialled during dressing changes and dialysis. Tissue viability nurses proposed the use of Entonox for dressing changes; this was unfortunately not an option on this particular base ward, although it may be considered in other cases. Despite all of this, pain remained an especially difficult component of the disease to manage in this case. Opioid use was further complicated by opioid-induced constipation, which was subsequently difficult to manage. The patient required multiple laxatives, including docusate sodium, senna, macrogols and lactulose. Some patients are also started on neuropathic analgesics, such as pregabalin, which have shown some benefit, reflective of the multifactorial nature of pain in calciphylaxis [8]. Additionally, management options were limited due to underlying renal impairment, which further complicated treatment options. Furthermore, severe pain, prolonged hospitalisation, functional decline and uncertainty regarding prognosis contribute to significant psychological distress, which can heighten pain perception and reduce coping ability. This results in pain that is often highly resistant to standard therapy, resulting in the need for early specialist pain involvement and an individualised, multimodal approach.

The use of corticosteroids for anti-inflammatory purposes in patients with calciphylaxis remains controversial. Corticosteroid therapy has been associated with increased risk of developing calciphylaxis, primarily through disturbances in calcium and phosphate metabolism, as a consequence of steroid-induced bone disease and degradation [12]. In addition, corticosteroids may suppress the immune system, hence predisposing patients to wound infections [13]. Conversely, a case-control study reported a positive and rapid clinical response following systemic corticosteroid administration in the majority of patients presenting with plaque-type calciphylaxis [5]. In this case, intravenous hydrocortisone was administered for five days when the patient became haemodynamically unstable. As she was not deemed a candidate for intensive care admission, systemic corticosteroid therapy was initiated as a salvage measure given the lack of alternative options to address her deteriorating clinical status. The patient demonstrated improvement in overall clinical condition afterwards.

Limited evidence is available regarding the most commonly isolated organisms from calciphylaxis-associated wounds. One study reported that gram-negative organisms were the predominant isolates, with *Pseudomonas aeruginosa* identified as the most prevalent species [14]. In contrast, wound swabs obtained from our patient on admission cultured two uncommon Gram-positive organisms, which posed additional challenges to antimicrobial management. As these isolates differed from the more typical gram-negative pathogens reported in wound infections, empirical therapy was less straightforward despite the guidance of the microbiology team. The presence of mixed and evolving microbial profiles obscured the initial clinical picture and complicated treatment decisions. Due to persistently elevated CRP levels following an initial course of intravenous teicoplanin and ertapenem, repeat wound cultures were performed, which subsequently yielded a gram-negative organism. Antimicrobial therapy was adjusted accordingly. Effective management of calciphylaxis-related wounds requires a multidisciplinary approach, and surgical intervention should be considered to optimise clinical outcomes [15]. Further research on common isolated organisms of calciphylaxis wounds would benefit patient outcomes in the future. 

Surgical debridement should be considered when feasible, as necrotic wounds are highly susceptible to infection, which may hinder overall clinical progress if not addressed accordingly [16]. The goal of surgical debridement is the removal of devitalized tissue while preserving surrounding viable tissue to promote optimal wound healing [17]. This procedure requires input from highly specialised surgeons. Evidence from previous studies indicates that patients with calciphylaxis who underwent surgical debridement demonstrated a significantly improved one-year survival rate of 61.6%, compared to 27.4% in those who did not undergo the procedure [12]. However, our patient was not deemed a suitable candidate for surgical debridement due to multiple comorbidities and the elevated risk of perioperative complications.

**Figure 5:**
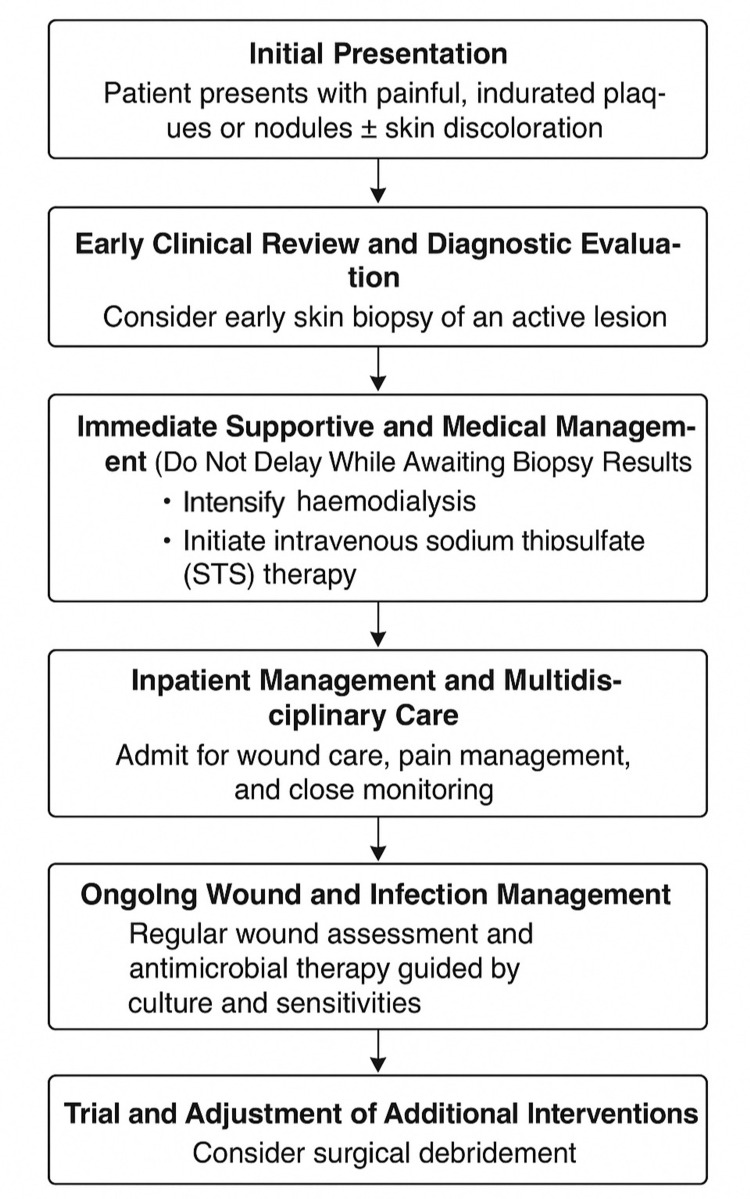
Recommended care pathway flowchart for calciphylaxis. Image credit: Ryan Ching Nam Lau

Early identification of calciphylaxis is essential for improving clinical outcomes, given the high associated mortality. Healthcare professionals should maintain a high level of suspicion in patients presenting with painful, non-healing cutaneous lesions, particularly when multiple risk factors are present, as in the patient in our case. These include ESRD on haemodialysis, high BMI, warfarin (vitamin K antagonists) use and exposure to corticosteroids. In this case, warfarin was replaced with apixaban, as warfarin inhibits the synthesis of matrix Gla protein (MGP), a vitamin K-dependent protein that normally prevents vascular calcium deposition; hence, its use may contribute to the promotion of vascular calcification [7]. In suspected cases of calciphylaxis, prompt initiation of intensified haemodialysis and administration of sodium thiosulfate should be considered, even before histopathological confirmation. A skin biopsy remains important to establish the diagnosis definitively and should be arranged without undue delay [1,7]. There is a critical need to enhance awareness among healthcare professionals, especially those in general practice and emergency medicine, as patients may initially present with non-specific symptoms. Thorough history-taking and clinical examination are key to recognising early signs, and urgent referral to nephrology and dermatology specialists is recommended to guide further investigation and management. Moreover, patient education plays a significant role in early detection. Individuals with chronic kidney disease, particularly those receiving dialysis, should be informed about calciphylaxis as a potential complication. They should be encouraged to report new or unusual skin lesions promptly, enabling earlier intervention. The goal is to improve the high morbidity and mortality associated with calciphylaxis through ensuring early recognition and timely multidisciplinary management. In summary, a care pathway schematic has been created to guide similar future practices (Figure 5).

## Conclusions

This case highlights the complex and multifactorial nature of calciphylaxis, as well as the significant challenges involved in its management. The patient presented with multiple well-known risk factors, and all these factors should prompt early clinical suspicion in similar scenarios. Despite appropriate escalation of care, the patient experienced ongoing calciphylaxis-related pain, wound infections, opioid-induced constipation and delirium. In summary, clinicians should maintain a high level of suspicion and consider biopsy in high-risk ESRD individuals presenting with painful, indurated skin lesions. Besides, healthcare professionals should also initiate STS therapy along with an escalated dialysis schedule while awaiting pathology. Pain management in calciphylaxis requires specialist input and careful balancing to avoid adverse effects. Nonetheless, timely and coordinated care involving the multidisciplinary team is essential. Furthermore, early patient education regarding symptom recognition and prompt presentation may play a critical role in improving outcomes. This case illustrates the aggressive nature of calciphylaxis and the importance of raising awareness among healthcare professionals and at-risk individuals, prompting early diagnosis and care management as illustrated in the care pathway schematic.

## References

[1] Jeong HS, Dominguez AR (2016). Calciphylaxis: controversies in pathogenesis, diagnosis and treatment. Am J Med Sci.

[2] Westphal SG, Plumb T (2022). Calciphylaxis. https://www.ncbi.nlm.nih.gov/books/NBK519020/.

[3] Kodumudi V, Jeha GM, Mydlo N, Kaye AD (2020). Management of cutaneous calciphylaxis. Adv Ther.

[4] Chinnadurai R, Huckle A, Hegarty J, Kalra PA, Sinha S (2021). Calciphylaxis in end-stage kidney disease: outcome data from the United Kingdom Calciphylaxis Study. J Nephrol.

[5] Fine A, Zacharias J (2002). Calciphylaxis is usually non-ulcerating: risk factors, outcome and therapy. Kidney Int.

[6] Bhambri A, Del Rosso JQ (2008). Calciphylaxis: a review. J Clin Aesthet Dermatol.

[7] Chang JJ (2019). Calciphylaxis: diagnosis, pathogenesis, and treatment. Adv Skin Wound Care.

[8] Chinnadurai R, Sinha S, Lowney AC, Miller M (2020). Pain management in patients with end-stage renal disease and calciphylaxis- a survey of clinical practices among physicians. BMC Nephrol.

[9] Cohen GF, Vyas NS (2013). Sodium thiosulfate in the treatment of calciphylaxis. J Clin Aesthet Dermatol.

[10] Gaisne R, Péré M, Menoyo V, Hourmant M, Larmet-Burgeot D (2020). Calciphylaxis epidemiology, risk factors, treatment and survival among French chronic kidney disease patients: a case-control study. BMC Nephrol.

[11] Udomkarnjananun S, Kongnatthasate K, Praditpornsilpa K, Eiam-Ong S, Jaber BL, Susantitaphong P (2019). Treatment of calciphylaxis in CKD: a systematic review and meta-analysis. Kidney Int Rep.

[12] Weenig RH, Sewell LD, Davis MD, McCarthy JT, Pittelkow MR (2007). Calciphylaxis: natural history, risk factor analysis, and outcome. J Am Acad Dermatol.

[13] Gökten DB, Mercan R (2025). Calciphylaxis: a mimic of casculitis. Mediterr J Rheumatol.

[14] Puca V, Marulli RZ, Grande R (2021). Microbial species isolated from infected wounds and antimicrobial resistance analysis: data emerging from a three-year retrospective study. Antibiotics (Basel).

[15] Tsolakidis S, Grieb G, Piatkowski A, Alharbi Z, Demir E, Simons D, Pallua N (2013). Calciphylaxis - a challenging &amp; solvable task for plastic surgery? A case report. BMC Dermatol.

[16] Pertea M, Benamor M, Bulgaru-Iliescu AI, Abid A, Abid S, Amarandei AH (2025). Calciphylaxis of the upper limbs: a rare and serious disease with multidisciplinary treatments—a case series and literature review. Diagnostics (Basel).

[17] Nigwekar SU, Kroshinsky D, Nazarian RM (2015). Calciphylaxis: risk factors, diagnosis, and treatment. Am J Kidney Dis.

